# Assessment of Canadian perinatal mental health services from the provider perspective: Where can we improve?

**DOI:** 10.3389/fpsyt.2022.929496

**Published:** 2022-09-23

**Authors:** Laurel M. Hicks, Christine Ou, Jaime Charlebois, Lesley Tarasoff, Jodi Pawluski, Leslie E. Roos, Amanda Hooykaas, Nichole Fairbrother, Michelle Carter, Lianne Tomfohr-Madsen

**Affiliations:** ^1^Renée Crown Wellness Institute, University of Colorado Boulder, Boulder, CO, United States; ^2^School of Nursing, University of Victoria, Victoria, BC, Canada; ^3^Orillia Soldiers' Memorial Hospital, Orillia, ON, Canada; ^4^Department of Health and Society, University of Toronto Scarborough, Toronto, ON, Canada; ^5^IRSET - Institut de Recherche en Santé, Environnement et Travail, University of Rennes 1, Rennes, France; ^6^Department of Psychology, University of Manitoba, Winnipeg, MB, Canada; ^7^College of Social and Applied Social Sciences, University of Guelph, Guelph, ON, Canada; ^8^Faculty of Medicine, University of British Columbia, Vancouver, BC, Canada; ^9^School of Nursing, University of British Columbia, Vancouver, BC, Canada; ^10^Department of Psychology, University of Calgary, Calgary, AB, Canada

**Keywords:** depression, mental health, postpartum, pregnancy, screening, treatment

## Abstract

**Purpose:**

Perinatal mental health disorders are common, and rates have increased during the COVID-19 pandemic. It is unclear where providers may improve perinatal mental health care, particularly in countries lacking national guidelines, such as Canada.

**Methods:**

A cross-sectional survey of perinatal health providers was conducted to describe the landscape of perinatal mental health knowledge, screening, and treatment practices across Canada. Providers were recruited through listservs, social media, and snowball sampling. Participants completed an online survey that assessed their perinatal mental health training, service provision types, their patient wait times, and treatment barriers, and COVID-19 pandemic-related impacts.

**Results:**

A total of 435 providers completed the survey, including physicians, midwives, psychologists, social workers, nurses, and allied non-mental health professionals. Most (87.0%) did not have workplace mandated screening for perinatal mental illness but a third (66%) use a validated screening tool. Many (42%) providers stated their patients needed to wait more than 2 months for services. More than half (57.3%) reported they did not receive or were unsure if they received specialized training in perinatal mental health. Most (87.0%) indicated there were cultural, linguistic, and financial barriers to accessing services. Over two-thirds (69.0%) reported the COVID-19 pandemic reduced access to services.

**Conclusion:**

Survey findings reveal significant gaps in training, screening tool use, and timely and culturally safe treatment of perinatal mental health concerns. There is critical need for coordinated and nationally mandated perinatal mental health services in Canada to improve care for pregnant and postpartum people.

## Introduction

Mental health disorders during the perinatal period (defined as conception through postpartum; PMHD) are the most prevalent health complications in pregnancy, affecting 10–25% of perinatal individuals (1–4). The risk of experiencing PMHDs is significantly higher in groups exposed to historical and current marginalization and stigmatization, including immigrant and Indigenous populations (5–7). When PMHDs are untreated, there is an increased risk of future mental health difficulties, reduced quality of life, and high healthcare utilization (8). Beyond the individual impact on the pregnant parent, perinatal mental illness confers a significant risks to children (9, 10) and the wider family system along with financial costs to society (11–13).

PMHD prevalence increased significantly during the COVID-19 pandemic. Estimate show significantly more pregnant people report clinical levels of depression and anxiety symptomatology with 37% reporting clinically relevant levels of depression and 57% reporting clinically relevant levels of anxiety (1, 3). Primary care or psychiatrist mental health visits also increased during the first 9 months of the COVID-19 pandemic, highlighting the increased pressure on services (14).

Negative effects of PMHDs can be mitigated with timely screening and subsequent treatment (15). Many health authorities recommend screening for PMHDs during pregnancy and postpartum using validated screening tools (16–18). Even though it is widely accepted that professionals should assess perinatal mental health regularly and there are brief, validated screening tools available, it is estimated that health care professionals identify only 25% of perinatal individuals with postpartum depression (PPD) and even less with other PMHDs (19). Concerningly, up to 70% of perinatal women will not seek treatment (20). As a result, only 15% of women with a PMHD will receive evidence-based care (21), and these rates are lower in marginalized groups (22) as well as with fathers and partners (23).

Canada does not currently have a national strategy for screening and treating PMHDs (24) and therefore we suspect there is a range of mental health care that is offered to individuals during the perinatal period. We suspect that professionals may not be screening, some may be using clinical judgment only and others may be using validated screening tools. Additionally, it is unknown what specific perinatal mental health training professionals have received and what treatments are readily available. To better understand the current landscape of perinatal mental health in Canada, including provider training, provision of screening and treatment, current barriers, and needs in the context of the COVID-19 pandemic (blinded for review), created, and administered an online survey for Canadian health care providers in the fall of 2020. The information obtained *via* this survey and analyzed in this manuscript provides important information on the standard of care for perinatal people experiencing mental illness in Canada and may provide information that can help improve mental health care.

## Methods

We conducted an online survey of health care providers of perinatal people in Canada. The study was approved by the (blinded for review). Individuals were eligible to participate in the survey if they were perinatal care providers (e.g., physicians, midwives, nurses, psychologists, social workers) or other perinatal professionals (e.g., doulas) in Canada. Participants were recruited *via* social media, email lists of perinatal service providers and organizations, and snowball sampling.

### Survey

We administered a 57-item online survey, in English and French, developed for the purposes of this study. Survey questions were predominantly multiple-choice format and asked about participant demographics, occupational role, population served, perinatal mental health training, perinatal mental health screening and treatment, and treatment barriers and access. Demographic items included participant age, gender, profession, and ethnicity. Rurality was defined based on workplace postal code. For many survey items, respondents could select more than one applicable choice.

Survey items were developed by a small group of (blinded) members, followed by iterative consultation and feedback process with the (blinded). The survey data were collected *via* Qualtrics (Provo, UT), a survey platform that is compliant with Canada's Freedom of Information and Protection of Privacy Act.

### Procedures

Participants consented prior to survey entry. Data collection occurred between October 5, 2020, and December 16, 2020. Participation was voluntary; participants did not receive monetary compensation.

### Analysis

Surveys were excluded if they did not consent, or did not live in Canada. Survey responses were optional and partial survey completers were included in the analysis. Descriptive statistics were generated. *T*-tests were used for testing comparisons between professions on perinatal mental health training. Prior to conducting the analyses, data were examined to verify quality and identify any potential univariate outliers (25). SPSS version 27 was used for statistical analyses (26). Unless otherwise indicated, missing data was present in < 5% of a given variable of interest with no further statistical tests of missingness performed.

## Results

### Participant characteristics

A total of 732 individuals met the study inclusion criteria and consented to participate; 435 completed the full survey. No outliers were identified. Respondents were predominantly female (95.6%), reported European ethnicity (81.5%), and had a median age of 31–40 years (41.5%). Providers from a wide variety of professional training backgrounds completed the survey (Table 1). We grouped providers by profession and training, based on presumed mental health and perinatal service provisions speciality. Professional categories included: midwives (16.1%), nurses (17.7%), social workers (9.4%), interdisciplinary mental health professionals (20.5%, including psychiatrists, psychologists, psychotherapists, counselors who did not hold other roles as nurses, social workers, or midwives), medical doctors from non-mental health specialties (19.5%, including obstetricians/gynecologists, pediatricians, family physicians, and internists), naturopathic doctors (13.4%), and allied non-mental health professionals (13.6%, including doulas, physiotherapists, chiropractors, and a registered dietician). We included psychiatrists in the interdisciplinary mental health professional category rather than with medical doctors as they frequently have much more specialized mental health training than other medical doctors in Canada. Most provider workplaces were in urban settings (94.1%) and provided perinatal mental health services locally (59.5%), with 13.9% also providing services online. Three-quarters of professionals (77.4%) provided services in English.

**Table 1:** Demographics and professional characteristics of survey participants.

**Characteristic**	**Values**	**Sample frequency (*N*, %)**	**Valid *n***
Gender	Female	412 (95.6)	431
	Male	16 (3.7)	
	Neither	3 (0.7)	
Age	20–30 years	52 (12.0)	434
Range 23–44 years (*M* = 32.6, *SD* = 3.77)	31–40 years	180 (41.5)	
	41– 50 years	129 (29.7)	
	51–60 years	46 (10.6)	
	61–70 years	22 (5.1)	
	>70 years	5 (1.1)	
Location—province	Ontario	207 (50.9)	407
	British columbia	78 (19.2)	
	Quebec	48 (11.8)	
	Alberta	38 (9.3)	
	Newfoundland & labrador	8 (2.0)	
	Nova scotia	8 (2.0)	
	Saskatchewan	7 (1.7)	
	Manitoba	4 (1.0)	
	New brunswick	4 (1.0)	
	Yukon territory	3 (.7)	
	Prince edward Island	1 (.2)	
	Nunavut/NW territory	1 (.2)	
Rural-based^a^	Yes	24 (5.9)	405
	No	381 (94.1)	
Ethnicity^b^	European	352 (81.5)	432
	Indigenous	26 (6)	
	African	10 (2.3)	
	South Asian	10 (2.3)	
	Middle eastern	9 (2.1)	
	Latin American	8 (1.9)	
	East Asian	6 (1.4)	
	Caribbean	4 (.9)	
	South East Asian	4 (0.9)	
	Oceanic	3 (0.7)	
Minority identification	Yes	52 (12.0)	434
	No	382 (88.0)	
Profession^b^	Physician	99 (22.8)	434
	Family doctor	65 (65.7)	
	OB-GYN	14 (14.1)	
	Psychiatrist	14 (14.1)	
	Pediatrician	2 (2.0)	
	Other	4 (4.0)	
	Nurse	77 (17.7)	
	Midwife	70 (16.1)	
	Naturopathic doctor	58 (13.4)	
	Doula	52 (12.0)	
	Social worker	41 (9.4)	
	Counselor	38 (8.8)	
	Psychotherapist	19 (4.4)	
	Psychologist	18 (4.1)	
	Chiropractor	3 (.7)	
	Physiotherapist	3 (.7)	
	Registered dietician	1 (.2)	
	Other	14 (3.2)	
Location of service provision^b^	Local	423 (59.5)	433
	Provincial	139 (19.5)	
	National	30 (4.2)	
	International	20 (2.8)	
	Online	99 (13.9)	
Language service provision^b^	English	336 (77.4)	434
	French & English	57 (13.1)	
	French	20 (4.6)	
	Other	21 (4.8)	
Training in perinatal mental health	Yes	185 (42.7)	433
	No or Unsure	248 (57.3)	

a Based off of workplace postal code;

b Participants were able select all applicable choices.

### Findings

#### Perinatal mental health training

Less than half of participants (42.7%) indicated they had PMHD specific training (Table 1). Of those who had specific training, 36.2% reported they had specific training experiences in residency or practicum, 18.5% reported they had completed a two-day Postpartum Support International training (www.postpartum.net/professionals), and 45.3% indicated “other” training, which included self-guided reading and continuing education.

**Figure 1:**
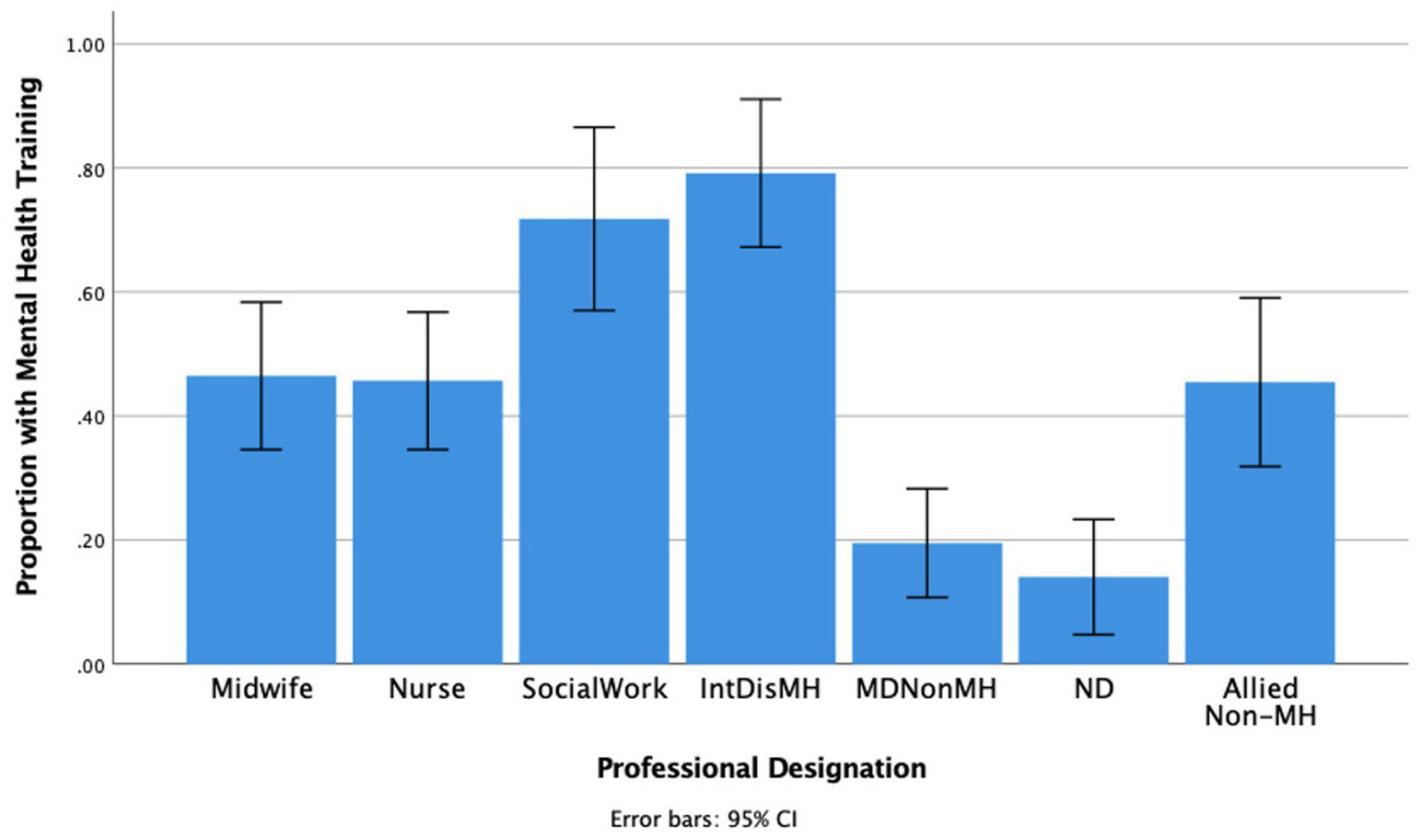
Perinatal Mental Health Specific Training by Profession. *IntDisMH* includes psychiatrists, psychologists, psychotherapists, and counselors and this is the group that we would expect would most likely screen for and treat PMHDs. *MDNonM*H, Obstetrician-gynecologists, family doctors, pediatricians, other physicians; *ND*, Naturopathic doctors; *Allied Non-MH*, Allied non-mental health professionals.

Prevalence of PMHD training background by provider type indicated notable differences by specialty (Figure 1) ranging from 79.2% for interdisciplinary mental health professionals (e.g., psychologists, psychotherapists, counselors, and psychiatrists) to 14.0% for naturopathic doctors (see Figure 1). Other than social work, all professions reported significantly lower perinatal specific mental health training compared to interdisciplinary mental health professionals (*t* = 3.74–8.71, all *p* < 0.001).

#### PMHD screening and validated tool use

Most (91.5%) providers indicated they screened patients for PMADS; however, only 66.0% of these providers reported using a validated screening tool (*n* = 287). When asked further detail about what tool they use, nearly half stated they use informal screening (47.7%) (e.g., used clinical impressions and asked questions if there was a clear concern, or asked about general wellbeing) as a valid method. Tools used varied widely, and included several validated measures, including the Edinburgh Postnatal Depression Scale (EPDS), Patient Health Questionnaire (PHQ-9), and General Anxiety Disorder-7 (GAD-7) (see Table 2).

**Table 2:** PMHD screening tools reportedly used by participants.

**Tool^a^**	***N* (%)**
Edinburgh postnatal depression scale	255 (88.9)
Informal screening^b^, no tool	137 (47.7)
Patient health questionnaire-9	70 (24.4)
General anxiety disorder-7	64 (22.3)
Patient health questionnaire-2	35 (12.2)
General anxiety disorder-2	34 (11.8)
Mood disorder questionnaire	14 (4.9)
Postpartum depression screening scale	11 (3.8)
Postpartum depression predictors inventory	4 (1.4)
Other^c^	28 (9.8)

*n* = 287.

a Participants were able select all applicable tools;

b Inquiring about a person's mental health without a screening tool;

c Included open-ended responses that contained other validated and non-validated tools.

Examining the use of validated tools by provider type indicated notable differences by specialty, with >75% of providers in interdisciplinary mental health professions, social work, midwifery, or nursing, using validated tools. In contrast < 45% of non-mental health speciality MDs (e.g., obstetricians, internal medicine) or NDs reported using a validated tool. Of allied non-mental health professions, 61.0% reported using a validated tool. Compared to midwives, with the highest reported rates of validated screening tools (87.3%), there were no significant differences in frequency by interdisciplinary mental health professions (83.0%), social work (76.9%), or nursing (76.5%). In contrast, MDs (43.4%), NDs (45.6%), and allied non-mental health professions (61.5%) all reported lower frequency of validated tools use, compared to midwives (*t*s range 3.46–6.31, *p*s = < 0.001).

Of the respondents who answered questions about screenings policies for PMADs, only 12.9% indicated that they were conducting mandated screening by their workplace.

#### Referrals, treatment, and accessibility

Most providers (89.4%) reported barriers to PMHD treatment (Table 3), with the most common being long wait times (80.9%), financial concerns (31.2%), and lack of culturally relevant services (30.7%). Other issues included local agencies only offering crisis care (under four sessions; 24.1%), lack of perinatal mental health specialists in the community (23.4%), language barriers (14.3%), no local resources available (12.1%), and “other” (30.2%), which included an overemphasis on pharmaceutical intervention compared to holistic approaches to care, lack of services for uninsured individuals, internalized stigma about mental illness, lack of transportation and childcare, and geographical barriers.

**Table 3:** Management for positive screens for mental health concerns reported by participants.

	**Types**	**Frequency (% of cases)^b^**	**Valid N**
Referrals sources^a^	Public	143 (35.5)	403
	Private	40 (9.9)	
	Both	245 (60.8)	
	Other	63 (15.6)	
Treatment protocol^a^	**Referral**		403
	Counseling/Psychotherapy	310 (76.9)	
	Pharmaceutical therapy	140 (34.7)	
	Further testing	120 (29.8)	
	**Initiated treatment**		424
	Counseling psychotherapy
	Supportive Counseling	168 (39.6)	
	Cognitive behavioral therapy (CBT)	92 (21.7)	
	Interpersonal therapy	39 (9.2)	
	Solution focused therapy	27 (6.4)	
	Eye movement desensitization and reprocessing (EMDR)	15 (3.5)	
	Screen and treat physical health conditions (e.g., anemia)	137 (32.3)	
	Pharmaceutical therapy	87 (20.5)	
	Other	131 (30.9)	
Treatment accessibility	Accessibility issues	380 (89.4)	425
	No accessibility issues	45 (10.6)	
Types of accessibility issues^a^	Long wait times	322 (80.9)	398
	No resources for non-maternal caregivers	130 (32.7)	
	Financial barriers	124 (31.2)	
	No culturally relevant options	122 (30.7	
	Only crisis care offered	96 (24.1)	
	No specialized PMH providers	93 (23.4)	
	Language barriers	57 (14.3)	
	No local resources	48 (12.1)	

a Participants were able select all applicable choices;

b Percentage reflects percentage/proportion of cases across all responses.

Just over one-third (35.5%) of respondents indicated they refer to a publicly-funded mental health provider, 9.9% refer to a private mental health provider, and the majority refer to both 60.8% (Table 3). A small percentage (15.6%) indicated they refer to “other,” which included patients' primary care provider or family physicians, Indigenous treatment centers, and community-based support groups. Several providers noted they referred based on a patient's insurance benefits coverage or socioeconomic status.

Nearly one-third of providers (32.7%) reported there were no available treatment options for non-maternal parents (e.g., fathers and non-birth parents).

#### Typical wait times for therapy

**Figure 2:**
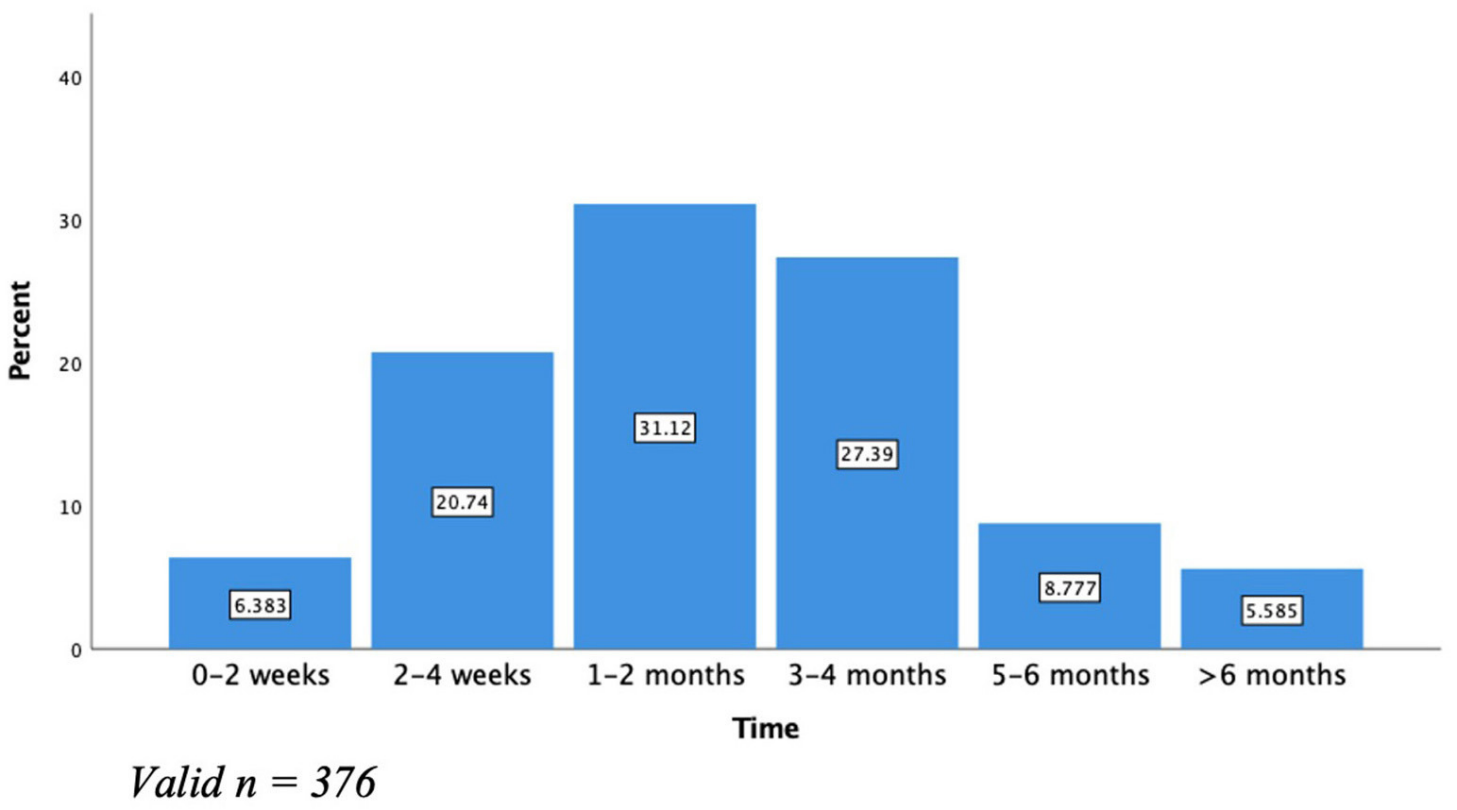
Wait times for perinatal mental health treatment. *Valid n* = *376*.

Twenty-seven percent of providers reported clients who could access a referral were able to receive therapy within a month, while most providers (67.3%) reported wait times between 1 and 6 months, and 5.6% reported wait times longer than 6 months (Figure 2).

#### Health equity and unmet needs

When asked, “do you think that perinatal and postpartum services are sensitive to the unique needs of people belonging to diverse backgrounds (i.e., racial/ethnic minority background, disabilities, new Canadians, minority sexual orientations and gender identities)?,” 38% responded “no,” 30.8% were “uncertain,” and 25.9% of providers responded “yes.” A small percentage (5.3%) indicated “other” (e.g., “Unlikely,” “Sometimes,” “Yes, but there is always room for improvement”). Most providers (87%) indicated that people of diverse backgrounds encounter barriers when accessing perinatal mental health services, including a lack of providers from diverse backgrounds, cultural insensitivity, and white-centric, hetero- and cis-normative systems that create barriers to access. Only 3.2% indicated that perinatal mental health services mostly meet existing needs. Providers reported that services in Canada only partially (93.5%) or were not (6.5%) meeting existing needs.

### The impact of the COVID-19 pandemic

Most providers (69.3%) indicated increased difficulty accessing services for their clients during the COVID-19 pandemic. At the time of the survey, Fall 2020, only 54.1% of providers reported that they had resumed their usual model of providing perinatal mental health care. Several providers noted how the shift to virtual care and the inability to conduct in-person groups and home visits had created challenges (e.g., increased feelings of isolation and anxiety, discomfort with assessments and treatment over the phone, lack of privacy and childcare options). Where in-person treatment was available, some commented on the challenges encountered, such as the inability of partners/spouses to accompany patients to appointments and masks impeding the ability of providers to read patients' expressions and to demonstrate empathy. Some reported that services and funding had completely ceased during the pandemic.

## Discussion

In this online survey-based study of perinatal mental health from the perspective of providers in Canada, we found gaps in training, screening, treatment, and accessibility. Despite PMHDs being the most common complication of the perinatal period (27), only 43% of providers indicated that they had received perinatal specific mental health training, with wide variation in the content, quality, and quantity of training. At this time, there is no specific perinatal mental health training requirement in Canada for an individual to treat and identify as a specialist in PMHD, Postpartum Support International has developed a certification process that any provider can apply for which ensures a minimum level of qualifications, including 2 years of practice, a 14 h training in PMHD, 6 h of advanced PMHD training and successfully passing a certification exam (https://www.postpartum.net/professionals/certification). One limitation of this study is that we allowed providers to self-report if they had perinatal mental health training without triangulating these data to ensure the training they received was of sufficient quality. Regardless of this, the fact that 43% of providers indicate any training at all shows there is significant room for improvement.

While we found that many providers reported screening for PMHDs, the definitions of “validated tools” varied with the majority using informal screening methods. It is important to mention that most validated screeners used in health care focus on depression screening and do not screen for other mood disorders such as perinatal anxiety, obsessive compulsive disorder, and psychosis. Numerous barriers to care were identified, including long wait times, with members of equity deserving groups experiencing the most barriers to access. Finally, providers reported the COVID-19 pandemic negatively impacted service delivery. Overall, providers across Canada resoundingly agreed that perinatal mental health services do not meet the current need. These findings suggest health care in Canada must improve drastically to support people in pregnancy and postpartum.

This is the first survey study assessing the state of perinatal mental health in Canada from the perspective of providers. Our findings are consistent with what has been found in other countries. Byatt, Simas (28) also found that most prenatal care providers report inadequate perinatal mental health training when looking at North American outpatient obstetric settings (28). Likewise, a survey of 223 American obstetricians and gynecologists found that lack of training was a barrier to routine screening for postpartum depression and postpartum psychosis (29). Both providers and patients in studies from other countries identified similar access barriers to perinatal mental health services. For example, a review of 35 qualitative studies in the UK identified poor knowledge of perinatal mental illness among providers, often attributed to inadequate training opportunities, unclear policies and inconsistent use of validated screening tools, in addition to barriers concerning language and differences in cultural values as barriers to providing and accessing perinatal mental health services (30). A recent review also found that midwives participating in studies in the European Union, UK, Australia and other countries, lacked perinatal mental health training and engage in inconsistent screening practices (31). Midwives identified lack of knowledge regarding the referral processes, long wait times, lack of specialized services, and uneven distribution of available resources geographically as barriers (31). Overall, the findings from our survey expand this research by describing the state of perinatal mental health care screening in Canada and further demonstrate a need to implement a standard of care for pregnant and postpartum people.

Our findings concerning the impact of the COVID-19 pandemic on access to perinatal mental health services are worrisome, as numerous studies (3), including those conducted in Canada (14), have found increased rates of PMHDs and perinatal mental health service use during the pandemic. As Vigod, Brown (14) argue, there is a need for effective and accessible perinatal mental health care as the pandemic continues.

### Limitations

Although our results provide much needed knowledge of the current conditions of perinatal mental health care in Canada, the key limitation is the use of convenience sampling and thus it is unclear if the survey reflects the current conditions across Canada. Future research is needed to understand differences in PMH training, screening, referral, and treatment practices between provinces and territories of Canada, as well as to identify access barriers specific to different parts of Canada.

Participation or non-response bias is a limitation to consider, as many survey respondents indicated they had some perinatal mental health training, which may not be representative of the Canadian health care provider population. We speculate this is a result of a high number of survey respondents having an affinity to the topic. Thus, more research is needed to understand the mental health training experiences of specific types of providers who work with pregnant and parenting people, including the content covered in said training, when or how often screening takes place along the perinatal period, who is responsible for screening, and what supports are available if the screening warrants more care.

### Implications

Our findings have important practice and policy implications. There is a need to create more accessible services specific to PMHDs and for populations who experience high rates of PMHDs, including those tailored to diverse and often-marginalized groups, such as racialized (e.g., Black) and Indigenous populations, immigrants, and people with disabilities, and numerous barriers to care (including stigma and socio-economic barriers), which may contribute to underuse of perinatal mental health services (32). Findings suggest a need for formal, standardized provider perinatal mental health training, particularly among physicians from non-mental health specialties, such as obstetricians and family physicians, who provide the majority of perinatal care in Canada (33). Our findings suggest a need for the development of consistent, evidence-based guidelines concerning perinatal mental health screening, referral, and treatment at a national level.

Guidelines should involve a commitment to integrated models of care and proactive collaboration across specialties (i.e., multidisciplinary perinatal care is vital) and clear guidance concerning which providers are responsible for screening at different points of care. In the early stages of the perinatal period, family physicians play a key role in screening and referral for PMHDs, whereas in the postpartum period, family physicians and pediatricians may play a key role in screening and referral for PMHDs (34). Those who provide much of the care during pregnancy and the early postpartum period (i.e., obstetricians, midwives) as well as professionals (i.e. social workers, psychologists, peer specialists, doulas) are needed in order to meet the needs of perinatal people (35). Pediatricians are often overlooked as the child is their patient, however there are strong practice guidelines that suggest a baby's well-visit appointment it is an opportune time for pediatricians screen the parent, and that by providing care to the parent, the infant's health can be affected in a positive way (36). Lack of specialized training for professionals and policies on screening, and timely referrals may have serious consequences for perinatal people and their families. For professionals, lack of training may contribute to lack of confidence and competence to address PMHD among patients and both lack of training and policies supporting mandated screening may impact quality of care, care access, and clinical outcomes.

This is also an opportunity to highlight the benefit of integrated behavioral health (37) and offering care from a variety of different types of providers. In an integrated behavioral health setting, it is possible to have individuals such as medical assistants to screen each individual, who can then communicate positive screening to medical doctors, who can refer and discuss options for treatment which may include psychopharmacology and evidence-based therapy with a Licensed Mental Health Professional. Additionally, offering services within the community, such as doula and peer support can be an effective way to engage individuals, such as racialized people, who are more likely to have a lack of trust in the medical systems. Many racialized communities prefer gaining their support from providers who are from their community and also from agencies that are within their community (38).

It is also important to note that training and screening are important, but they alone are not enough to ensure the wellness of perinatal individuals. Byatt, Xu (39) recommend mental health pathways that include a full spectrum of services and follow-up beginning with screening, assessment, triage and referral, ensuring treatment access, treatment initiation, and then ensuring there is symptom monitoring and responsive treatment adaptations based on quantitative data until symptoms remit.

It is reported that obstetric providers who are not trained in PMHD are less likely to screen for and/or discuss mental health concerns (28). Conversely, universal screening may contribute to better outcomes, greater service utilization, and reduction of stigma around perinatal mental illness (37); in fact, in countries where screening is mandated, not only do more perinatal people get screened but screening alone contributes to improved outcomes (40). Further, a cross-sectional study of formal and informal mental health screening of pregnant women in Alberta found that most viewed screening as a positive experience and many reported that screening made them feel their provider cared about them (41), countering perceptions that screening itself is psychologically harmful.

## Conclusion

The providers who completed this survey overwhelmingly reported that perinatal mental health services across Canada are insufficient. Study findings indicate gaps in perinatal mental health training, screening, and service provision, especially for diverse and often-marginalized groups. Although services for perinatal people have expanded overtime, and professionals understand much more about the mental health of perinatal people compared to 50 years ago, there is still significant room for improvement. Given high rates of PMHD, which have increased during the COVID-19 pandemic, there is an urgent need to address the many gaps identified; more perinatal mental health training, greater use of validated screening tools, and greater accessibility of services, especially services that are financially accessible and culturally safe. Unlike countries such as Australia (24), Canada does not have a national perinatal mental health strategy. The findings of this survey support a growing body of evidence that a national perinatal mental health strategy, including investment in training and treatment, is vital to better support the mental health of pregnant and parenting people and their families across Canada.

## Data availability statement

The original contributions presented in the study are included in the article/supplementary material, further inquiries can be directed to the corresponding author.

## Ethics statement

The studies involving human participants were reviewed and approved by University of Calgary REB. The patients/participants provided their written informed consent to participate in this study.

## Author contributions

LH contributed to all part of the manuscript, led the manuscript completion, the conceptualization and creation of the survey, recruitment, data collection, data analysis, and manuscript preparation. CO contributed to survey development, recruitment, data collection, data analysis, and manuscript preparation. JC and AH contributed to survey development, data collection, recruitment, and manuscript preparation. LT contributed to recruitment, data collection, and manuscript preparation. JP and NF contributed to recruitment, and manuscript preparation. LR contributed to recruitment, data analysis, and manuscript preparation. LT-M contributed to the survey development, research methodology, data analysis, and manuscript preparation. All authors contributed to the article and approved the submitted version.

## Conflict of interest

The authors declare that the research was conducted in the absence of any commercial or financial relationships that could be construed as a potential conflict of interest.

## Publisher's note

All claims expressed in this article are solely those of the authors and do not necessarily represent those of their affiliated organizations, or those of the publisher, the editors and the reviewers. Any product that may be evaluated in this article, or claim that may be made by its manufacturer, is not guaranteed or endorsed by the publisher.
